# Stereo-cell coupled with single-cell transcriptomics identifies a transitional metabolic state in maturing skeletal muscle myofibers

**DOI:** 10.3389/fgene.2026.1846707

**Published:** 2026-06-17

**Authors:** Langchao Liang, Yuxin Gong, Chaochao Chai, Shijie Hao

**Affiliations:** 1 School of Biology and Biological Engineering, South China University of Technology, Guangzhou, China; 2 State Key Laboratory of Genome and Multi-omics Technologies, BGI Research, Hangzhou, China; 3 Key Laboratory of Spatial Omics of Zhejiang Province, BGI Research, Hangzhou, China; 4 Key Laboratory of Brain Cell Mapping of Zhejiang Province, BGI Research, Hangzhou, China; 5 College of Life Science and Technology, Huazhong Agricultural University, Wuhan, Hubei, China

**Keywords:** metabolism, myofiber, scRNA, snRNA, stereo-cell

## Abstract

Identification of skeletal muscle fiber types and metabolic reprogramming are crucial for postnatal muscle maturation, but high-resolution metabolism and spatial heterogeneity of muscle fibers in mid-maturation remain poorly understood. Our study performed single-cell RNA sequencing (scRNA-seq) and single-cell nuclear RNA sequencing (snRNA-seq) on five hind limb muscles from 5-week-old mice, combined with Stereo-cell at single muscle fiber resolution, to elucidate myofiber subtype maturation and its metabolic changes. Integrating scRNA-seq and snRNA-seq data, a comprehensive mouse skeletal muscle cell atlas was constructed, demonstrating the complementary advantages of the two techniques in capturing interstitial cells and multinucleated muscle fibers. Our analysis resolved a continuous maturational lineage from type I to IIB myonuclei (IIB_1–3). Notably, the IIB_3 myonuclei subtype exhibited a dual hypermetabolic phenotype, with increased oxidative phosphorylation (OXPHOS) and glycolytic activity, which differs from the purely glycolytic phenotype observed in adult mice. Stereo-cell further validated this transitional metabolic state and revealed spatial heterogeneity within individual IIB myofibers, with localized high-oxidation regions. Furthermore, we observed a mixed myofiber phenotype, with subtype-specific myosin heavy chain expression enriched at the myofiber terminals, indicating directional transformation of myofiber during maturation. In summary, this study reveals a previously undescribed transitional metabolic feature of IIB-type myofiber during postnatal muscle maturation and elucidates the spatial metabolic heterogeneity of myofiber, providing new insights into the regulatory mechanisms of skeletal muscle developmental plasticity and metabolic specialization.

## Introduction

Skeletal muscle serves as the core effector organ responsible for movement and energy metabolism in the body. Its complex physiological functions highly dependent on the cellular heterogeneity of myofibers (Schiaffino and Reggiani, 2011; Serrano et al., 2023). Classic histomorphometric analyses—including muscle wet weight, length, cross-sectional area, myofiber cross-sectional area (CSA), Feret’s diameter, and fiber density—together with immunohistochemical (IHC) typing based on myosin heavy chain (MyHC) isoforms, have established the gold standard for characterizing muscle architecture and fiber type composition (Battey et al., 2024). Based on myosin heavy chain isoform expression, contractile kinetic characteristics and energy metabolic patterns, mammalian skeletal muscle myofibers are classified into four classic subtypes: type I (slow-twitch, expressing MyHC I encoded by *Myh7*); type IIA (fast-twitch oxidative-glycolytic, MyHC IIa/*Myh2*); type IIX (fast-twitch intermediate, MyHC IIx/*Myh1*); and type IIB (fast-twitch glycolytic, MyHC IIb/*Myh4*) (Schiaffino and Reggiani, 2011; Qaisar et al., 2016). Type I myofibers are rich in mitochondria and rely primarily on oxidative phosphorylation for energy supply, exhibiting sustained endurance contraction characteristics. Type II myofibers contract more rapidly, among which type IIB myofibers display highly activated glycolytic metabolic pathways and specialize in rapid, explosive limb movements, while types IIA and IIX exhibit an intermediate metabolic phenotype of oxidative-glycolytic metabolism (Schiaffino and Reggiani, 2011). Different muscles in the hind limbs of mice exhibit natural subtype composition differences. The soleus muscle (SOL) is dominated by type I slow-twitch myofibers, whereas the extensor digitorum longus (EDL) and tibialis anterior (TA) are highly enriched in type IIB fast-twitch myofibers. This feature makes it a classic ideal model for studying myofiber subtype differentiation and metabolic specialization (Ham et al., 2022).

The functional specialization of myofiber subtypes is essentially determined by the precise regulation of metabolic phenotypes. The establishment of specific metabolic programs, as a core event in myofiber identity construction, directly shapes the energy supply patterns and functional adaptability of different subtypes (Hostrup and Deshmukh, 2025; Schiaffino et al., 2007). Recent studies have clearly shown that the differential activation of oxidative phosphorylation (OXPHOS) and glycolytic pathways is a key metabolic marker distinguishing myofiber subtypes (Pereyra et al., 2022; Monzel et al., 2023; Murgia et al., 2021; Egan and Zierath, 2013). Furthermore, the type I to type IIB myofiber transition stands is one of the most representative directions in the field of skeletal muscle fiber plasticity research. Existing studies have confirmed that this transition is accompanied by prominent metabolic reprogramming. Under pathophysiological conditions such as long-term immobilization, denervation, or obesity, slow-twitch type I myofibers gradually transform into fast-twitch type IIB myofibers. The core metabolic hallmarks of this conversion encompass reduced mitochondrial content, downregulated expression of key oxidative phosphorylation (OXPHOS) enzymes, and concurrently upregulated expression of core genes in the glycolytic pathway. Ultimately, this leads to a complete transition from an oxidative phenotype to a pure glycolytic phenotype (Zhang et al., 2024; Lafontan and Langin, 2009). However, conventional transcriptomic approaches—including bulk sequencing, single-cell RNA-seq (scRNA-seq), and single-nucleus RNA-seq (snRNA-seq)—dissect myofiber heterogeneity at the expense of spatial information. These methods cannot resolve the intrafiber spatial heterogeneity of multinucleated myofibers, such as regionalized metabolic domains or directional subtype conversion along the fiber axis, severely limiting our understanding of myofiber maturation in its native spatial context.

Notably, postnatal skeletal muscle maturation in mice involves a critical window characterized by myofiber type specialization, metabolic reprogramming, and contractile apparatus maturation, spanning from 3 to 7 weeks after birth (Simon et al., 2025; White et al., 2010). Most existing studies focus on adult, fully mature myofibers, systematically elucidating the classic differentiation paradigm that slow-twitch myofibers rely on oxidative metabolism and type IIB fast-twitch myofibers are dominated by glycolysis (Schiaffino and Reggiani, 2011; Bourdeau Julien et al., 2018). Although previous studies have suggested myofiber subtype conversion and metabolic remodeling during the postnatal stage, most investigations focus on global transcriptional changes or early developmental nodes, lacking high-resolution dissection of the dynamic characteristics of mid-maturation, incompletely committed myofibers (Simon et al., 2025; Giacomello et al., 2020). In particular, the process by which type IIB myofibers—the most abundant and functionally critical subtype—gradually establish the adult metabolic phenotype, and the temporal switching of oxidative phosphorylation and glycolytic pathways during maturation, remain poorly defined.

To overcome these long-standing technical limitations, we leverage stereo-cell, a recently developed spatial enhanced-resolution single-cell sequencing platform built on high-density DNA nanoball (DNB) arrays (Liao et al., 2025).

Unlike droplet-based microfluidic systems, Stereo-cell requires no compartmentalized encapsulation and is uniquely compatible with large, multinucleated, and structurally complex cells such as skeletal myofibers. It enables *in situ* transcriptome profiling at single-myofiber resolution, preserves the spatial localization of transcripts and myonuclei, supports imaging-guided cell segmentation, and allows unbiased quantification of gene expression within morphological intact myofibers. This technology thus fills the critical gap between single-nucleus transcriptomics and spatial tissue profiling, providing an unprecedented tool to map transitional metabolic states and spatial heterogeneity within individual maturing myofibers.

Therefore, this study selected 5-week-old postnatal mice as the model, systematically collecting the hindlimb soleus (SOL), extensor digitorum longus (EDL), tibialis anterior (TA), gastrocnemius (GAS), and quadriceps (QUAD). We constructed a high-resolution myofiber subtype atlas using single-cell and single-nucleus transcriptome sequencing. Through pseudotime analysis and metabolic pathway signature scoring, we not only finely delineated the maturation trajectory of myofibers from type I to IIA, IIX, and IIB, but also discovered that type IIB myofibers in 5-week-old mice exhibit a transitional metabolic phenotype with dual high levels of oxidative phosphorylation (OXPHOS) and glycolysis, rather than the pure glycolytic phenotype typical of adulthood. More importantly, using Stereo-cell, we achieved *in situ* validation of this transitional metabolic state, and revealed the existence of localized high-oxidative metabolism domains within individual type IIB myofibers as well as directional subtype conversion enriched at myofiber termini. This discovery provides a novel perspective for dissecting the dynamic regulation of metabolic plasticity during skeletal muscle maturation.

## Methods

### Collection of mouse muscle samples and ethics

A total of four male 5-week-old C57BL/6 mice were used in this study: 1 mouse for scRNA-seq, a second mouse for snRNA-seq, and two additional mice for Stereo-cell (one for EDL dissection, one for SOL dissection). All experimental procedures were approved by the Institutional Review Board/Ethics Committee of BGI (approval No. BGI-IRB A24039) and conducted in accordance with local legislation and institutional requirements. The tibialis anterior (TA), extensor digitorum longus (EDL), gastrocnemius (GAS), soleus (SOL), and quadriceps (QUAD) were dissected from each hindlimb of the respective mice.

### Preparation of single-cell (nucleus) suspension

Different muscle tissues were minced into approximately 2 mm fragments and digested with 2.5% collagenase II (Sigma-Aldrich, catalog number C6885-1G) and collagenase IV (Sangon Biotech, catalog number A004186-0001) in a 37 °C water bath for 30 min. The digested cell suspension was sequentially filtered through 100 μm and 40 μm strainers in a stepwise manner, followed by centrifugation. The cell pellet was resuspended to obtain a single-cell suspension. For single-nucleus isolation, fresh muscle tissue rather than the above single-cell suspension was used as the starting material. Nuclei were isolated using ATAC wash buffer, followed by centrifugation and resuspension to generate a single-nucleus suspension. The quantity and viability of single-cell (nucleus) suspensions were assessed using AOPI staining.

### Single-cell library construction and sequencing

Library preparation was performed using the DNB C-series Single-Cell Library Preparation Kit (MGI, 1000021082) according to the manufacturer’s instructions. Approximately 20,000 cells (nuclei) were loaded onto each C4 scRNA chip; mRNA hybridization with capture beads was performed at room temperature for 20 min. Droplets were disrupted to collect beads, and reverse transcription was performed in a Veriti thermal cycler. After the reaction, products were collected for cDNA PCR amplification, followed by sorting and purification of cDNA and Oligo products. After concentration determination, cDNA and Oligo libraries were constructed and sequenced on the BGISEQ-T10 platform: the cDNA library was sequenced with a 47 + 100 + 10 configuration, and the Oligo library with a 32 + 42 + 10 configuration.

### Raw data quality control

All sequencing data preprocessing and quality control were performed using the MGI single-cell transcriptome-specific tool DNBC4tools v2.1.1. First, sequence parsing was performed on raw Oligo and cDNA reads to accurately extract cell barcodes (CB) and unique molecular identifiers (UMI), laying the foundation for single-cell sample discrimination and correction of PCR amplification bias. Subsequently, read-level QC was conducted to filter low-quality bases with Phred scores <20, adapter sequences, and abnormal reads with excessive N content, ensuring a Q30 qualification rate ≥90% for base calling accuracy. Clean cDNA reads after read-level QC were aligned to the mouse reference genome GRCm39 and annotated for genes. UMI deduplication was applied to correct PCR amplification bias and achieve accurate quantification of gene expression, ultimately generating a high-quality single-cell gene expression matrix. The R package DoubletFinder v2.0.3 was used to filter doublets at a 5% ratio for each library. Based on the interquartile range (IQR) method, cells were filtered by the number of detected genes (nFeature_RNA) and UMI counts (nCount_RNA): thresholds were set as Q3 + 1.5 × IQR and Q1 − 1.5 × IQR, retaining cells with both indices within the threshold range. Cells with mitochondrial gene ratio (percent.mt) > 30% (damaged cells) were also filtered. After multi-dimensional QC, 90,600 high-purity, high-reliability single cells were obtained for subsequent downstream analysis.

### Cell type annotation

The R package Seurat V4 was used for QC and clustering of the filtered matrix (Hao et al., 2021). Cell clusters were annotated based on widely reported cell type-specific molecular markers and functional enrichment analysis (Supplementary Table S1).

### Identification of differentially expressed genes

The “FindAllMarkers” function in the Seurat package was used to assess gene expression differences between target cell clusters and other clusters *via* the Wilcoxon rank-sum test. Only upregulated genes were screened, with “min.pct” and “logfc.threshold” set to 0.25. Significant differentially expressed genes with adjusted p-value (p_val_adj) < 0.05 after multiple testing correction were retained. Ribosome-related genes, mitochondrial genes, and specific non-coding RNAs were further filtered to eliminate interference from non-functional genes.

### GO enrichment analysis

Functional enrichment analysis of DEGs was performed using the R package clusterProfiler (Xu et al., 2024), with the mouse genome annotation database org. Mm.e.g.,.db as the reference (Gentleman et al., 2004). Significantly enriched Gene Ontology (GO) terms were defined as those with adjusted p-values <0.05, to elucidate the biological functions of each gene set.

### Monocle3 pseudotime analysis

Developmental trajectory analysis was conducted using the R package monocle3 (Cao et al., 2019). The Seurat object was converted to a “cell_data_set” object, retaining the previously computed UMAP coordinates for consistency. Cell clustering was performed based on the UMAP embedding, and a developmental trajectory graph was constructed using the “learn_graph” function. Cells annotated as “MF_I” were selected, and the vertex closest to each cell on the main trajectory was identified. The most frequent vertex was set as the root node, and pseudotime values for all cells were calculated using the “order_cells” function. Pseudotime distribution was visualized using the ‘plot_cells’ function.

### AddModuleScore

Pathway enrichment scores for glycolysis and oxidative phosphorylation (OXPHOS) were calculated using the “AddModuleScore” function in Seurat. A custom gene set was defined for glycolysis, while OXPHOS genes were retrieved from the org. Mm.e.g.,.db database using the GO term GO:0006119. Sample annotations were reclassified by skeletal muscle type (EDL, GAS, QUAD, SOL, TA). “AddModuleScore” was applied to compute pathway enrichment scores per cell, which involved normalization, binning, and averaging of gene expression values within each pathway. Scores were then Z-score normalized to eliminate batch and expression-level differences, and mean scores were calculated for each muscle fiber subtype.

### Stereo-cell workflow

#### Extraction of myofiber suspension

Dissociated EDL and SOL were immediately transferred to centrifuge tubes, supplemented with 5 mL digestion buffer containing 0.2% collagenase I, and digested in a 37 °C water bath for 30–60 min, with gentle mixing every 10 min. After 30 min, muscle tissue was examined microscopically; digestion was terminated when tissue edges were loose and myofiber bundles were exposed. Digested muscle was transferred to a culture dish with 10 mL washing buffer (90% DMEM + 5% FBS + 0.05% sodium pyruvate). A Pasteur pipette was used to gently rinse the muscle surface to release single myofibers until approximately 50 myofibers were obtained. Myofibers were transferred to a clean culture dish, washed with washing buffer, and then transferred to a PBS-containing culture dish (2–3 transfers) to remove residual DMEM and avoid interference with subsequent procedures. All myofibers were aspirated from PBS and transferred to a methanol-modified 6 cm culture dish with a total volume of ∼1 mL. While gently shaking the dish, 1 mL pre-chilled methanol was added (forming a 50% methanol mixture), and fixation was performed at −20 °C for 3 min. After fixation, 1 mL liquid was removed from the PBS-methanol mixture, 1 mL methanol was added again, and fixation continued for 3 min (75% methanol). This step was repeated twice until the methanol concentration approached 100%, and the myofiber suspension was stored on ice for later use.

#### Stereo-cell technical procedure

A wide-bore tip was used to aspirate 30–50 μL of myofibers and spread them on the chip, with sufficient volume aspirated in one step; if insufficient, excess methanol was removed, and an equal volume of myofibers was added. Residual liquid was aspirated, and myofiber positioning was adjusted *via* methanol pipetting, followed by air-drying. DAPI staining (Beyotime Biotechnology, catalog number C1006) was performed for 5 min, followed by washing with 0.1×SSC, air-drying, and addition of glycerol to cover the chip for bright-field and DAPI fluorescence imaging. Permeabilization, pre-hybridization, and reverse transcription (RT) reactions were performed: permeabilization and pre-hybridization reagents were prepared on ice, equilibrated to room temperature before permeabilization, and permeabilized at room temperature for 30 s. Pre-hybridization was performed with 20% formamide at room temperature for 5 min, followed by addition of RT reagent to the chip and incubation at 42 °C for ≥3 h. For library release, amplification, and purification: chips were transferred to 24-well plates, supplemented with 400 μL cDNA Release Mix per chip, sealed, and incubated at 55 °C for 3–18 h. Released products were collected *via* pipetting, and a second elution was performed with NF-H_2_O. The eluate was incubated with VAHTSTM DNA Clean Beads at a 1:0.7 ratio for 10 min at room temperature, washed with 80% ethanol, air-dried, and resuspended in NF-H_2_O. PCR amplification was performed in two tubes (42 μL sample +58 μL PCR Mix per tube). Amplified product concentration was detected and recorded; cDNA was purified *via* bead binding, ethanol washing, and NF-H_2_O elution, followed by concentration and fragment size analysis. Qualified cDNA was constructed into sequencing libraries and sequenced on the BGISEQ-T10 platform with a 47 + 100+10 configuration.

#### Myofiber identification

Stereo-cell raw gem files were processed using R language. The data. table package was used to read files and extract coordinates of each spatial locus, which were binned at 80 μm resolution (coordinate values divided by 80 and rounded). A sparse gene expression matrix was constructed using the Matrix package (rows: gene ID, columns: binned cell units, values: MIDCount), and a Seurat object was created using the CreateSeuratObject function in the Seurat package. Coordinate information of cell units was integrated into the object metadata and saved as an RDS file. After obtaining the nCount heatmap of stereo-cell data, it was imported into ImageJ (version 1.54p, National Institutes of Health, Bethesda, MD, USA) for manual outlining of myofiber contours, and mask files of all myofibers were exported containing coordinate information of the outlined regions. Based on the myofiber mask files annotated by ImageJ, the DBSCAN density clustering algorithm (eps = 5, minPts = 10) was used to identify intact myofiber regions, assign a unique fiber_id to each myofiber, and remove clustering noise points. Pixel coordinates of mask files were precisely matched with spatial coordinates of gem files, retaining overlapping loci.

Each myofiber was divided into ROIs: according to the original Stereo-cell protocol (Liao et al., 2025), each ROI contained 6,400 unique DNB loci. The total DNB count of single myofibers was calculated to determine the number of clusters (k = total DNB count/6,400). For non-integer k values, results were rounded to the nearest integer, and k was set to a minimum of one to ensure every myofiber had at least one ROI. K-means clustering was used to divide single myofibers into k ROIs, with local roi_local_id and global roi_global_id assigned to each ROI. Centroid coordinates of each ROI were calculated and matched to binned cell units in the Seurat object. Fiber_id, roi_global_id, and locus count were integrated into the Seurat object metadata. No myofiber exclusion criteria were applied; all morphologically intact myofibers identified by DBSCAN clustering were included for analysis. Cell units containing only ROI centroid coordinates were filtered to generate a myofiber-specific Seurat object for subsequent spatial transcriptomic analysis.

#### RCTD deconvolution

Stereo-cell spatial transcriptomic data was performed using the RCTD (Robust Cell Type Decomposition) algorithm implemented in the R package spacexr (Cable et al., 2022). A single-cell transcriptomic dataset of MF_IIB muscle fiber subtypes was used as the reference, and spatial transcriptomic data was preprocessed accordingly. Gene expression matrices were extracted for both the single-cell reference and spatial data, with genes split into 100 equal groups for batch-wise extraction and merging. Cell type annotations (single-cell), spatial coordinates (spatial data), and UMI counts (both datasets) were also extracted. A “Reference” object (containing single-cell expression matrix, cell type labels, and UMI counts) and a “SpatialRNA” object (containing spatial coordinates, spatial expression matrix, and UMI counts) were constructed. An RCTD model was created and run in multi-doublet mode to infer cell type composition and abundance of MF_IIB subtypes within spatial transcriptomic spots, and results were saved for downstream analysis.

#### Statistical analysis

Statistical differences in pathway scores between muscle fiber subtypes were analyzed using a two-tailed independent samples t-test. Normalized glycolysis pathway enrichment scores were used as the analytical variable, comparing MF_IIB_2 and MF_IIB_3 subtypes. To control for false discovery rate, Benjamini–Hochberg FDR correction was applied for multiple testing adjustment. The test was performed at a significance level of α = 0.05, with t-statistics and adjusted p-values calculated to quantify the significance of differences in glycolysis pathway activity between the two subtypes.

## Results

### Single-cell and single-nucleus RNA sequencing deciphers the cellular atlas characteristics of skeletal muscle in 5-week-old mice

**FIGURE 1:**
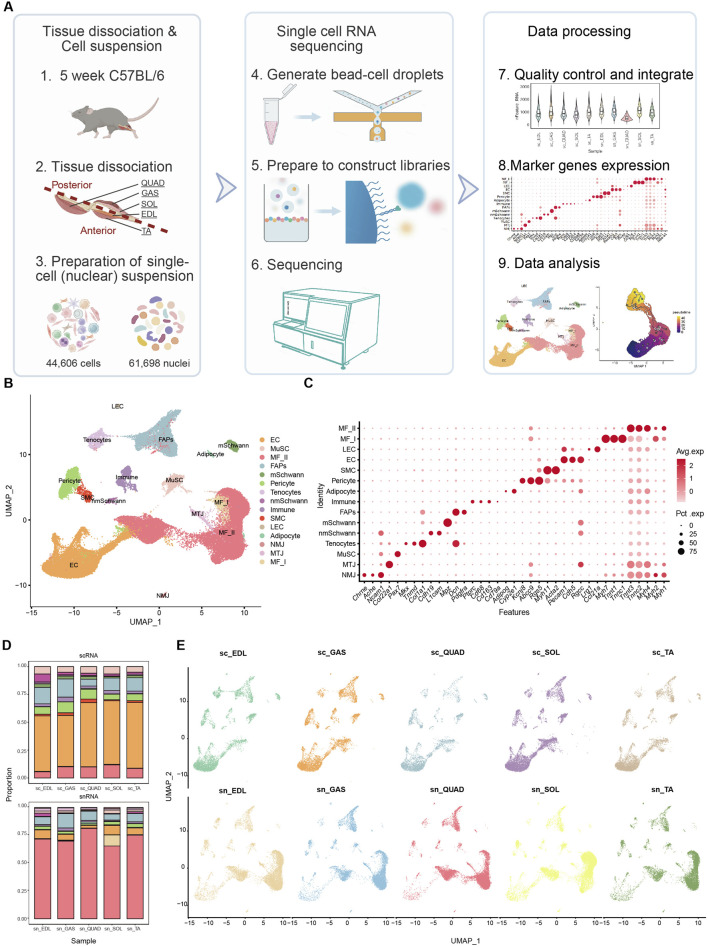
Generation of a single-cell transcriptomic atlas of mouse skeletal muscle **(A)** Schematic of the single-cell/nucleus RNA-seq workflow for mouse skeletal muscle. **(B)** UMAP visualization of cell type annotations from scRNA-seq of mouse skeletal muscle. **(C)** Expression of marker genes across cell types. Color intensity represents expression level, and bubble size indicates the proportion of cells expressing the gene. **(D)** Proportions of cell types across different sequencing technologies and skeletal muscles. **(E)** UMAP distribution of cells across different sequencing technologies and skeletal muscles.

To systematically analyze the cellular composition and transcriptomic characteristics of mouse skeletal muscle during the critical postnatal maturation window, we performed scRNA-seq and snRNA-seq on skeletal muscle tissues from 5-week-old C57BL/6 mice, and established a complete experimental workflow and data analysis system (Figure 1A). Through rigorous quality control of this workflow, we ultimately obtained high-quality sequencing data for 44,606 single cells and 61,698 single nuclei.

We performed unified manifold approximation and projection (UMAP) dimensionality reduction clustering analysis on the integrated scRNA-seq and snRNA-seq data, and annotated the cell clusters with a curated panel of lineage-specific marker genes to construct a transcriptome atlas of mouse hind limb skeletal muscle (Figure 1B; Supplementary Table S1). We identified multiple intrinsic cell types in mouse skeletal muscle, including muscle satellite cells (MuSC), fibro-adipogenic progenitors (FAPs), endothelial cells (EC), lymphatic endothelial cells (LEC), smooth muscle cells (SMC), immune cells, adipocytes, myelinating Schwann cells (mSchwann), non-myelinating Schwann cells (nmSchwann), tenocytes, pericytes, type I myofibers (MF_I), type II myofibers (MF_II), neuromuscular junction cells (NMJ), and myotendinous junction cells (MTJ). Each cell type formed independent and well-defined cell clusters in the UMAP space, indicating significant transcriptional heterogeneity among different skeletal muscle cell populations. Furthermore, each cell type expressed its canonical marker genes, confirming the reliability of cell clustering and annotation in this study and defining the core transcriptional features of each skeletal muscle cell type (Figure 1C).

By integrating cell proportion data and integration efficiency of each library from scRNA-seq and snRNA-seq, we found that sc and sn sequencing captured distinct cell types: scRNA-seq captured more ECs, pericytes, MuSCs, and mSchwann cells, while snRNA-seq captured most myofibers (MF), NMJ, and MTJ (Figures 1D,E). Consistent with previous reports, scRNA-seq excels at capturing small, easily dissociated cells, whereas snRNA-seq is superior for capturing large, multinucleated, and fragile cells (Chemello et al., 2020; Dos Santos et al., 2020; Tabula Muris Consortium, 2020; Petrany et al., 2020). Thus, the combination of these two technologies enables the construction of a more comprehensive and unbiased cellular atlas of mouse skeletal muscle. scRNA-seq better delineates the vascular microenvironment, stem cell, and immune cell states, while snRNA-seq provides in-depth dissection of myofiber heterogeneity and their specialized junctional structures.

We compared the cell type proportion characteristics of five major mouse skeletal muscle tissues in the scRNA-seq and snRNA-seq datasets (Figure 1D; Supplementary Tables S2, S3). We observed specific differences in cellular composition across skeletal muscle tissues: type I myofibers (MF_I) were almost exclusively distributed in the soleus (SOL) and rarely detected in other skeletal muscles, a finding that has been validated. The proportion of fibro-adipogenic progenitors (FAPs) in the gastrocnemius (GAS) was higher than in other skeletal muscles, a previously unreported observation that may indicate a stronger interstitial remodeling capacity or a more sensitive surveillance system for mechanical damage in GAS. The proportions of other cell types showed no significant differences across all skeletal muscle tissues. These results suggest that while the basic components of mouse skeletal muscle (e.g., endothelial cells, pericytes) are uniformly distributed, the proportions of functional units (MF) and key supportive cells (FAPs) determine muscle specificity.

### Single-nucleus transcriptomic atlas and metabolic heterogeneity analysis of mouse skeletal muscle myofibers

**FIGURE 2:**
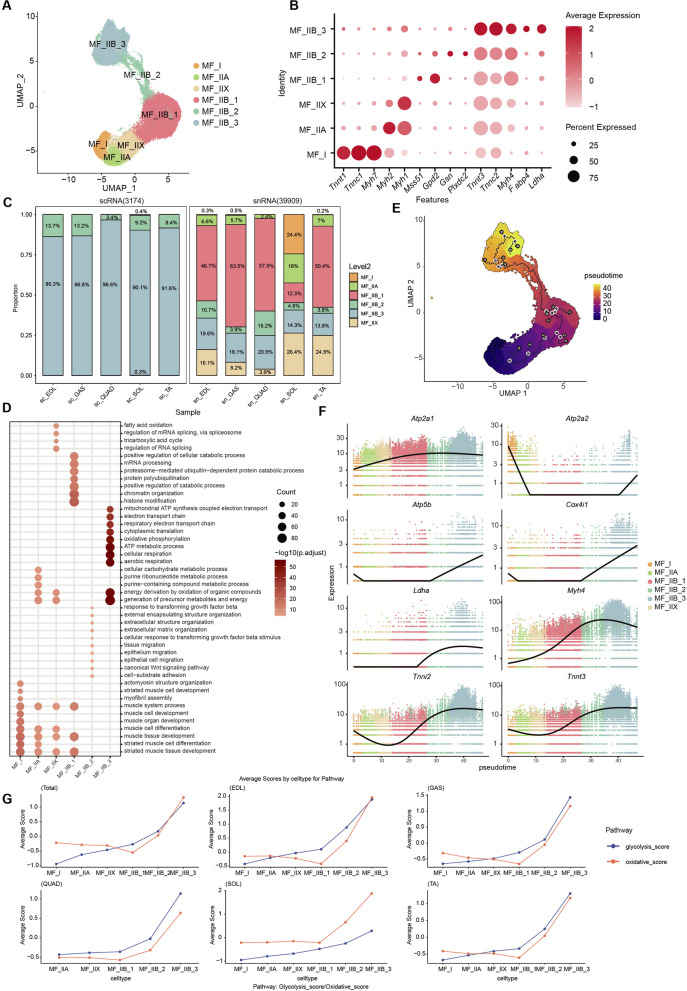
Transcriptomic features, developmental trajectories, and metabolic pathway differences among muscle fiber subtypes. **(A)** UMAP visualization of muscle fiber subtype annotations. **(B)** Expression of marker genes across muscle fiber subtypes. Color intensity represents expression level, and bubble size indicates the proportion of cells expressing the gene. **(C)** Proportions of muscle fiber subtypes across single-cell and single-nucleus sequencing data from different skeletal muscles. **(D)** Bubble plot of GO enrichment analysis for DEGs across muscle fiber subtypes. **(E)** Developmental trajectory and pseudotime analysis of mouse skeletal muscle fiber subtypes. **(F)** Dynamic expression of key functional genes across pseudotime in different muscle fiber subtypes. **(G)** Differences in glycolysis and oxidative phosphorylation pathway scores across muscle fiber subtypes in the total dataset and individual skeletal muscles.

We re-clustered myofiber data to systematically dissect myofiber heterogeneity. UMAP dimensionality reduction analysis subdivided myofibers into multiple subtypes, covering a continuous lineage from MF_I to MF_IIB (further subdivided into MF_IIB_1, MF_IIB_2, MF_IIB_3) (Figure 2A). MF_I fibers highly expressed *Tnnt1*, *Tnnc1*, and *Myh7*; MF_IIA fibers highly expressed *Myh2*; MF_IIX fibers highly expressed *Myh1*; and MF_IIB fibers highly expressed *Myh4* (Figure 2B). Comparison of scRNA-seq and snRNA-seq data revealed that scRNA-seq captured almost only a small number of MF_IIB_2 and MF_IIB_3. The SOL contained a high proportion of fast-twitch IIA and IIX myofibers, while other muscle tissues were dominated by the IIB subtype, with our data predominantly derived from myonuclei (Figure 2C; Supplementary Tables S4,5). Importantly, these snRNA-seq-derived myofiber subtype proportions agree closely with classical immunohistochemical fiber type distributions reported in the literature (Augusto et al., 2004; Bloemberg and Quadrilatero, 2012), confirming that our transcriptional fiber typing accurately recapitulates the known histological composition. Gene Ontology (GO) enrichment analysis of marker genes for each subtype (Figure 2D) showed that MF_I/IIA/IIX/IIB_1 myonuclei were enriched for functions related to muscle development and structural formation, with a decreasing number of enriched terms, implying the progressive specialization of myofiber subtypes during development and function. Interestingly, the MF_IIB_3 myonuclear subtype was enriched for the oxidative phosphorylation pathway, while its marker gene encodes a key glycolytic enzyme that catalyzes the reversible conversion of pyruvate to lactate (Sharma et al., 2022), suggesting that MF_IIB_3 myonuclei in 5-week-old mice may suggest a concurrent upregulation of both glycolytic and oxidative metabolism machineries.

Pseudotime analysis reconstructed the differentiation trajectory of myofibers from MF_I to MF_IIB (Figure 2E) and identified a series of key regulatory factors dynamically changing with the differentiation process. We found that in addition to myofiber subtype marker genes (e.g., *Myh4*, *Tnnt3*) whose expression increased with pseudotime, most other dynamically regulated genes were associated with metabolism. *Ldha* was barely expressed at the early pseudotime stage and gradually increased from MF_IIB_1. *Atp5b* encodes the catalytic subunit of mitochondrial ATP synthase, a terminal key protein of oxidative phosphorylation (OXPHOS) (Jonckheere et al., 2012). *Cox4i1* encodes a subunit of mitochondrial cytochrome c oxidase, the terminal key enzyme of the electron transport chain (Vogt et al., 2024). The expression of *Atp5b* and *Cox4i1* first decreased and then increased with pseudotime, peaking in MF_IIB_3, consistent with the enrichment of oxidative phosphorylation in MF_IIB_3 (Figure 2F). This dynamic expression pattern provides a new perspective for understanding the molecular mechanisms of myofiber metabolic specialization.

To further dissect the metabolic characteristics of myofiber subtypes, we performed metabolic pathway activity scoring for each subtype. The results showed that across all muscle tissues, glycolytic metabolism gradually increased from MF_I to MF_IIB_3. Oxidative metabolism gradually decreased from MF_I to MF_IIB_1, then increased thereafter, reaching the highest level in MF_IIB_3. These results confirm that skeletal muscle in 5-week-old mice remains in a developmental state, harboring myofiber subtypes with a dual-high state of glycolysis and oxidative metabolism.

### Stereo-cell reveals the dynamic conversion of myofiber subtypes in skeletal muscle

**FIGURE 3:**
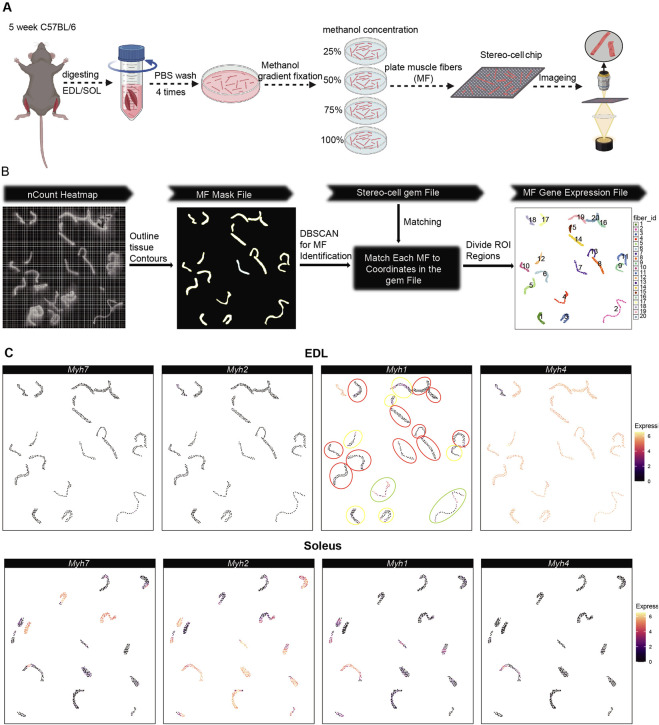
Spatial distribution of muscle fiber subtypes in mouse skeletal muscle revealed by Stereo‐cell **(A)** Experimental workflow for capturing single muscle fibers using Stereo‐cell. **(B)** Image processing pipeline to generate a gene expression matrix of muscle fiber ROIs. **(C)** Spatial expression distribution of muscle fiber subtype marker genes in EDL and SOL muscles. In the EDL *Myh1* panel, colored ellipses mark three expression patterns in *Myh4*+ (type IIB) fibers (n = 19): red (no/low *Myh1*, 57.9%), yellow (terminal enrichment, 31.6%), green (diffuse expression, 10.5%).

To dissect the molecular characteristics of skeletal muscle subtypes at single myofiber resolution, we established an expression profiling system combining single myofiber isolation from mouse extensor digitorum longus (EDL) and soleus (SOL) with Stereo-Cell technology (Figures 3A,B). After obtaining intact single myofibers via collagenase digestion, gradient methanol fixation was performed to preserve morphology and stabilize RNA, followed by spreading myofibers on Stereo-Cell chips for nucleic acid capture and imaging. Single myofibers were identified from nCount heatmaps using the DBSCAN algorithm and precisely matched with Stereo-Cell coordinate files; regions of interest (ROI) were segmented to generate a gene expression matrix at the single myofiber level (see Methods for details).

Expression analysis of myosin heavy chain isoform genes showed that the EDL was predominantly composed of MF_IIB myofibers, and *Myh1* (a marker of MF_IIX) exhibited a trend of expression at both ends of some MF_IIB fibers (6/19, 31.6%), consistent with previous reports (Liao et al., 2025). Intriguingly, we identified additional *Myh1* expression patterns in MF_IIB fibers, including complete absence of *Myh1* expression (11/19, 57.9%) or scattered *Myh1* expression not restricted to the fiber ends (2/19, 10.5%). These patterns may reflect heterogeneity in the transition from IIX to IIB fibers during maturation, possibly representing intermediate states of fiber type conversion (Figure 3C). In the SOL, the major subtypes were MF_I and MF_IIA. Similarly, we found that some MF_I myofibers expressed *Myh2* (a marker of MF_IIA) at both ends, potentially representing hybrid states of MF_I to MF_IIA conversion. These findings further confirm the dynamic conversion of myofiber subtypes in 5-week-old mouse skeletal muscle and reveal molecular heterogeneity at the resolution of a single myofibrillary fiber.

### Stereo-cell reveals high oxidative metabolism regions in type IIB myofibers

**FIGURE 4:**
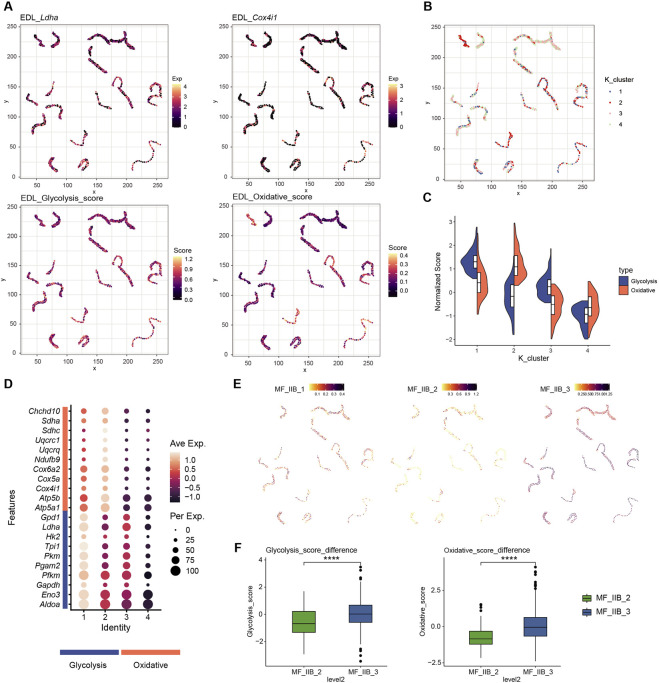
Oxidative phosphorylation and glycolysis exhibit distinct metabolic partitioning along MF_IIB muscle fibers **(A)** Spatial distribution differences in glycolysis and oxidative phosphorylation pathway gene expression along muscle fibers. **(B)** K-means clustering of muscle fiber ROIs based on metabolic pathway scores. **(C)** Heterogeneity in glycolysis and oxidative phosphorylation metabolic activity across clusters. **(D)** Expression differences in key glycolysis and oxidative phosphorylation pathway genes across clusters. **(E)** RCTD deconvolution of MF_IIB subtypes in Stereo-cell data. **(F)** Statistical differences in oxidative phosphorylation and glycolysis pathway scores between MF_IIB subtypes. *p < 0.05, **p < 0.01, ***p < 0.001,****p < 0.0001.

To further explore the metabolic state of MF_IIB myofibers, we used Stereo-cell data to visualize the expression of *Ldha* and *Cox4i1*, and performed signature scoring using glycolysis and oxidative phosphorylation gene sets (Figure 4A). The results showed that *Ldha* was more highly expressed across the entire myofiber, but there were also regions with high *Cox4i1* expression, and these regions exhibited higher oxidative metabolism scores. To identify these regions with high oxidative metabolism, we performed K-means clustering based on glycolysis and oxidative phosphorylation scores, dividing myofiber regions into four clusters (Figure 4B). Statistical analysis of glycolysis and oxidative phosphorylation scores for each cluster identified Cluster 2 as the high oxidative metabolism region (Figure 4C). Expression of key marker genes for these two pathways further validated this finding (Figure 4D). Subsequently, we projected single-cell myonuclear subtypes onto the entire myofiber, revealing distinct spatial localization preferences of different myonuclear subtypes (Figure 4E). MF_IIB_3 myonuclei exhibited the highest deconvolution scores and were distributed throughout almost the entire myofiber. MF_IIB_2 myonuclei showed preferential localization at the fiber terminals or in the central region. MF_IIB_1 myonuclei, with the lowest deconvolution scores (range 0–0.4), were scattered sporadically along the myofiber without obvious spatial clustering. These results demonstrate that even within a single myofiber of the same type (IIB), myonuclear subtypes are not randomly mixed but exhibit spatially organized functional domains. RCTD scoring of each region was used to annotate the corresponding myonuclear subtypes, and statistical analysis of metabolic pathway scores in each region showed that both glycolysis and oxidative phosphorylation scores were higher in MF_IIB_3 than in MF_IIB_2 (Figure 4F). This finding not only validated our previous hypothesis of the dual-high metabolic state of the MF_IIB_3 subtype, but also provided new evidence for understanding the spatial regulatory mechanisms of metabolic plasticity during skeletal muscle development.

## Discussion

In the conventional understanding of skeletal muscle biology, type IIB myofibers are regarded as the archetype of glycolytic metabolism, with extremely low mitochondrial content and oxidative phosphorylation levels (Reisman et al., 2024). However, during postnatal maturation, muscle fibers undergo dynamic metabolic remodeling, with age-dependent shifts in oxidative and glycolytic capacities (Giacomello et al., 2020). Through single-nucleus transcriptomic analysis of 5-week-old mice, this study discovered that the MF_IIB_3 myonuclear subtype in the critical maturation window exhibits a unique transcriptional signature of dual-high expression of genes related to glycolysis and oxidative phosphorylation (OXPHOS). This finding fills the temporal gap in metabolic remodeling during skeletal muscle development. We speculate that 5 weeks of age, a critical transition from juvenile to adult in mice, represents an “overlap phase” where myofibers are switching from early oxidative metabolism to the adult metabolic phenotype. This transitional metabolic program not only provides sufficient ATP supply for rapid myofiber hypertrophy but also reflects the precision of transcriptional regulation during development: the establishment of metabolic characteristics may not be an “on-off” switch, but a continuous and progressive remodeling process. We acknowledge a recent study that profiled metabolic changes in tibialis anterior muscle fibers across different ages in C57BL/6J mice, highlighting a transitional phase in early postnatal stages (Giacomello et al., 2020). Our findings at 5 weeks of age align with and extend this observation by revealing the transcriptional underpinnings of this transitional state at the myonuclear subtype level. Future studies are needed to address: when and by what signaling mechanisms does this dual-high state transition to the single glycolytic phenotype in adulthood? Is this transition driven by the maturation of neural electrical activity patterns, changes in circulating hormone levels, or an intrinsic myofiber developmental clock?

As typical multinucleated syncytia, whether skeletal muscle myofibers exhibit functional compartmentalization has long been a focus of research in the field. Using Stereo-cell technology at single myofiber resolution, this study observed that even within a single type IIB fiber, key oxidative metabolism genes are not uniformly distributed, but form distinct “high oxidative metabolism regions”. This indicates the existence of “myonuclear specialization domains” within myofibers, where specific myonuclei locally regulate surrounding cytoplasmic components to meet distinct physiological demands. Single-cell transcriptomic studies have revealed significant transcriptional heterogeneity within fast-twitch myofibers, and single-nucleus RNA-seq has further uncovered myonuclear subpopulations occupying distinct metabolic states (Petrany et al., 2020; Moiseeva et al., 2023; Peng et al., 2022; Giordani et al., 2019). A recent review further discusses the expanding knowledge about myonuclear heterogeneity, emphasizing that myonuclei may specialize their transcriptional output according to their spatial locale and functional demands, a concept directly supported by our spatial mapping of IIB_1, IIB_2, and IIB_3 myonuclei within single myofibers (Borowik et al., 2024). There is also single-cell evidence supporting the notion that myonuclear subpopulations within the same myofiber type may occupy distinct metabolic states (Verma et al., 2021). Such spatially localized metabolic regulation may be closely linked to the local microenvironment of myofibers, providing direct spatial evidence for understanding how multinucleated cells coordinate complex functions. Furthermore, *in situ* analysis of single myofibers *via* Stereo-cell technology revealed that *Myh1* (a marker of type IIX) is frequently enriched at both ends of type IIB fibers; this phenomenon is also observed during the conversion of type I to type IIA fibers in the SOL. This spatial expression pattern suggests that myofiber subtype conversion or maturation may be directional, proceeding from the fiber ends (near the myotendinous junction, MTJ) toward the central region. Given that skeletal muscle undergoes significant mechanical tension during growth and development, with the fiber ends serving as key stress concentration sites, mechanosensitive signaling pathways may preferentially trigger the switching of contractile protein isoforms. Stereo-cell technology not only validates previous hypotheses regarding the existence of “hybrid fibers” (Medler, 2019), but also reveals the physical trajectory of transcriptomic remodeling within mid-maturation myofibers at the *in situ* level, providing a novel perspective for dissecting the triggering mechanisms of skeletal muscle plasticity. Recent reviews have also summarized the broader landscape of spatial transcriptomic technologies applied to skeletal muscle, highlighting their power to reveal spatially defined cellular interactions that are lost in conventional single-cell approaches (Virtanen et al., 2025). Our Stereo-cell analysis applied this principle at the single-myofiber scale and further revealed directional myofiber conversion at the fiber ends, extending the reach of spatial transcriptomics into the study of multinucleated cell specialization.

This study constructed a multi-dimensional analytical framework for skeletal muscle cellular heterogeneity by integrating single-cell transcriptomics, single-nucleus transcriptomics, and Stereo-cell. This technical combination effectively overcomes the limitations of single sequencing technologies: scRNA-seq captures abundant interstitial microenvironment components, snRNA-seq solves the challenge of dissociating large multinucleated myofibers, and Stereo-cell restores the spatial logic of transcripts at single myofiber resolution. This progressive research strategy—from “single cell/single nucleus” to “*in situ* spatial resolution”—not only enables precise capture of the intermediate developmental state of skeletal muscle in 5-week-old mice, but also provides a referable technical approach for future studies of cell-cell interactions during muscle development and regeneration. A large-scale integration of single-cell transcriptomic data from mouse skeletal muscle has demonstrated the power of combining multiple datasets to capture rare transitional states in myogenesis (McKellar et al., 2021). Extending such integrative strategies to spatial multi-omics across developmental time points would further elucidate the dynamic regulatory logic underlying myofiber metabolic specialization. However, this study has certain limitations. First, although our conclusions are supported by cross-technology validation across multiple animals, each individual transcriptomic method was applied to a limited number of mice. This sample size is consistent with prior exploratory skeletal muscle scRNA-seq studies (Uapinyoying et al., 2023; Saleh et al., 2022). Nevertheless, future studies with larger biological replicates across both sexes are necessary to fully capture inter-individual variability and generalize the transitional metabolic phenotype. Second, although we identified the MF_IIB_3 myonuclear subtype with a dual-high metabolic state at the transcriptional level, transcriptional enrichment does not directly reflect metabolic pathway flux or enzymatic activity. High-throughput synchronous validation at the protein level and *in situ* metabolite distribution has not been performed due to technical constraints. Future studies should integrate spatial proteomics and spatial metabolomics to verify the physiological contribution of this transitional metabolic phenotype at the protein and metabolic levels. Third, one technical limitation of our Stereo-cell analysis is the modest number of intact myofibers obtained from EDL (n = 19) and SOL (n = 1 for IIB fibers), which is constrained by the current throughput of high-resolution spatial transcriptomic platforms for large multinucleated cells. Although our quantitative data (Figure 3C) support a directional conversion model, larger-scale studies with increased myofiber yield across multiple animals will be required to establish statistical robustness and generalizability.

## Data Availability

The data that support the findings of this study have been deposited in the China National GeneBank Sequence Archive (CNSA) under accession number CNP0008577. The code used in this study is deposited in GitHub and is available at https://github.com/lianglangchao/Skeletal-Muscle.
